# Mills’ syndrome revisited

**DOI:** 10.1007/s00415-019-09186-3

**Published:** 2019-01-10

**Authors:** Stephan R. Jaiser, Dipayan Mitra, Timothy L. Williams, Mark R. Baker

**Affiliations:** 1 Institute of Neuroscience, Medical School, Newcastle University, Framlington Place, Newcastle upon Tyne, NE2 4HH UK; 2 Department of Neurology, Royal Victoria Infirmary, Queen Victoria Road, Newcastle upon Tyne, NE1 4LP UK; 3 Department of Neuroradiology, Royal Victoria Infirmary, Queen Victoria Road, Newcastle upon Tyne, NE1 4LP UK; 4 Department of Clinical Neurophysiology, Royal Victoria Infirmary, Queen Victoria Road, Newcastle upon Tyne, NE1 4LP UK

**Keywords:** Mills’ syndrome, Spastic hemiparesis, MRI, Neurophysiology

## Abstract

Mills’ syndrome is an idiopathic, slowly progressive, spastic hemiparesis. We describe three cases that have been under review for a minimum of 11 years (range 11–19). In all patients, symptoms started in a leg, with a mean age of onset of 59 years (range 53–63). The only abnormality on laboratory investigations was a mildly elevated CSF protein in one case. MRI demonstrated focal T2 hyper-intensity located eccentrically in the cervical cord ipsilateral to the symptomatic side. No cerebral abnormality was demonstrated. Whilst visual and somatosensory evoked potentials were unremarkable, motor evoked potentials were abnormal in all patients: central motor conduction times were significantly prolonged unilaterally in two patients and bilaterally but asymmetrically in the third. Beta-band (15–30 Hz) intermuscular coherence, a potentially more sensitive method of assessing upper motor neuron integrity, was absent unilaterally in one patient and bilaterally in the other two. One patient developed amyotrophy and thus a picture of amyotrophic lateral sclerosis after 16 years, suggesting that Mills’ syndrome is part of the motor neuron disease spectrum. Both amyotrophy and subclinical contralateral upper motor neuron disease can therefore be features of Mills’ syndrome. However, even with the most sensitive electrodiagnostic techniques, unilateral upper motor neuron disease can remain the only abnormality for as long as 10 years. We conclude that whilst Mills’ syndrome should be classified as a motor neuron disorder, it is a distinct nosological entity which can be distinguished from amyotrophic lateral sclerosis, upper motor neuron-dominant amyotrophic lateral sclerosis and primary lateral sclerosis. We propose diagnostic criteria for Mills’ syndrome, and estimate a point prevalence of at least 1.2:1,000,000 based on our well-defined referral population in the North of England.

## Introduction

A case of idiopathic, slowly progressive, ascending, spastic hemiparesis was first described in 1900 by Charles Mills [21]. Post-mortem findings from a subsequent patient, who had been symptomatic for 8 years and died of tuberculosis, showed degeneration of the crossed and uncrossed pyramidal tracts originating from the left motor cortex, but no Betz cell degeneration within the left precentral gyrus or attrition of anterior horn cells [23]. By 1906, Mills had gathered a further seven cases of the syndrome, including one case where symptoms began in the arm, leading to the additional epithet ‘unilateral descending paralysis’ [22].

Mills’ assertion that the syndrome represents a separate clinical entity—distinct from amyotrophic lateral sclerosis (ALS), primary lateral sclerosis (PLS) and multiple sclerosis – has remained controversial [20]. Despite the limited investigations available at the time, two of the ten patients in Mills’ original case series received alternative diagnoses (neurosyphilis/taboparesis and multiple sclerosis), with one further case excluded by concomitant optic atrophy and cranial nerve palsies. Twenty-six cases of Mills’ syndrome have been reported since 1906, mostly as single case reports, but only 16 of these conformed to Mills’ original description of an idiopathic, isolated, progressive, spastic hemiparesis (Table 1). Of the remainder, two cases were diagnosed with ALS and a further two with multiple sclerosis [20], one evolved into PLS and also developed an extrapyramidal syndrome [33], one was attributed to lacunar infarcts [6], and two were ascribed to frontotemporal dementia/motor neuron disease [3, 8]. In two further cases, the presentation involved significant cognitive or neuropsychiatric abnormalities [10, 19].

**Table 1:** Summary of published cases conforming to Mills’ original description of an idiopathic isolated progressive spastic hemiparesis

References	Sex, mean age of onset (range)	Site of onset (*n*)	Sensory/autonomic features in paretic limb (*n*)	Atypical features (*n*)	Laboratory investigations (*n*)	Radiology (*n*)	NCS/EMG (*n*)	EP (*n*)	Mean FU (range)	Deaths (*n*), age, CoD; autopsy/histology
[22]	3M/4F 42 years (13–52)	R LL (2) L LL (2) R UL/LL (2) L UL (1)	Pain (1) Hyperaesthesia (1)	Vert. (1)	NR	NR	NR	SEP: NR VEP: NR MEP: NR	6 years (1.5–15)^a^	Yes (1), 60 years, CCF; bilateral but asymmetrical CST degeneration, no degeneration of Betz cells (1)
[20]	2M 64 years, 36 years	L LL (1) L UL (1)	NR	NR	CSF: NAD (2)	CT head: ipsilateral parietal CVA (1); NAD (1) Myelogram: NAD (1)	NCS: NAD EMG: PSW LL (1) UL/LL (1)	SEP: NR VEP: NR MEP: NR	6 years, 9 years	No
[13]	1M 6 years	R LL	NR	GCN	Blood NAD Urine NAD CSF NAD	CT head: NAD MRI brain: T2 hyperintensity of L pontine CST	NAD	SEP: NAD VEP: NAD MEP: NR	14 years	No
[14]	2F 25 years, 49 years	L UL R LL	No	Urinary urgency (1)	Syphilis (–) SPEP NAD Auto-Ab (–) CSF: NAD (1); OCB (–)/WBC < 3/ Protein 0.6 g/l (1)	MRI brain: NAD (2) MRI spine: NAD (2)	NAD (2)	SEP: NAD (2) VEP: NAD (2) MEP: NR	18 years, 17 years	No
[27]	1M 57 years	R LL	No	WM	SPEP: IgM lambda monoclonal band CSF NAD	MRI brain: SVD MRI spine: NAD	NCS: NAD EMG: FP + Fib + PSW in R UL/LL	SEP: NR VEP: NR MEP: NR	12 years	No
[33]	2M 40 years, 39 years	R UL/R LL (1) L LL (1)	No	No	CSF: protein 0.65 g/l (1), protein normal with no OCB (1)	MRI brain: NAD (1); 2 WMLs (1) MRI spine: NAD (1); high intensity at C3/4 + T1 (1)	EMG: NR (1), NAD (1)	SEP: bil. LL absent, UL normal (1), NR (1) VEP: NAD (1), L delay (1) MEP: L UL + LL ↑CCT (1), NR (1)	20 years, 3 years	No
[25]	1M 53 years	L LL	No	No	HIV (–) HTLV I–II (–) FTA/VDRL (–) SPEP NAD CSF NAD	MRI spine: NAD MRI brain: FLAIR/T2 hyperintensities R corona radiata + PTs bil	NCS: NAD EMG: Fib+PSW bilat UL/LL Fib + PSW bil UL/LL	SEP: NR VEP: NR MEP: NR	3 years	No
[28]	1M 20 years	R LL	No	Symptom onset after #R LL	HIV (–) HTLVI-II (–) CSF NAD	MRI brain: NAD MRI spine: NAD	NAD	SEP: NAD VEP: NR MEP: R UL + LL ↑CCTs	26 years	No
[11]	1M 40 years	R LL	Impaired vibration sense but near-normal proprioception	Weight loss	B12 NAD Syphilis (–) T4 21 (10–19), TSH NAD	CT head: atrophy, SVD CT cervical spine: degenerative changes but no neural compromise	NAD	SEP: NR VEP: NAD MEP: NR	35 years	Yes, 80, pneumonia; vacuolation, gliosis and demyelination of anterolateral cord; loss of anterior horn cells; no inflammation; Betz cells normal
[18]	3F 67 years (55–76)	R UL (2) R LL (1)	No	No	Genetics for SOD1, TARDBP, FUS, C9ORF72 (–)	MRI brain: normal (3) PET: hypometabolism in contralateral Rolandic and peri-Rolandic areas (3)	Delayed signs of LMN involvement (3)	SEP: NR VEP: NR MEP: NR	4.7 years (2–8)	No
[30]	1M 53 years	L LL	No	Impaired cognitive flexibility, borderline phonemic fluency	CSF NAD Genetics for SOD1, TARDBP, FUS, C9ORF72 (–)	MRI brain and cervical spine: NAD, incl. voxel-based morphometry PET: hypometabolism in bil. motor and premotor areas, larger on right	NAD	SEP: NR VEP: NR MEP: L UL + LL ↑CCT, R LL borderline ↑CCT	10 years	No
[34]	1M 17 years	L UL	No	No	B12 NAD Syphilis (–) HIV (–) CSF NAD	MRI head: atrophy of right cerebral peduncle and pontine base MRI cervical spine: NAD	NAD	SEP: NR VEP: NR MEP: NR	3 years	No

# fracture, *Auto-Ab* auto-antibodies, *bil*. bilateral, *C9ORF72* chromosome 9 open reading frame 72, *CCF* congestive cardiac failure, ↑*CCT* increased central conduction times, *CoD* cause of death, *CST* corticospinal tract, *CVA* cerebrovascular accident (stroke), *Dipl*. diplopia, *Fib* fibrillation potentials, *FLAIR* fluid attenuation inversion recovery, *FP* fasciculation potentials, *FTD* frontotemporal dementia, *FU* follow-up, *FUS* fused in sarcoma, *GCN* giant cutaneous naevus, *HTLV* human T-lymphotropic virus, *L* left, *LL* lower limb, *n/a* not applicable, *NAD* no abnormality detected, *NoR* no response, *NR* not reported, *Nyst*. nystagmus, *OA* optic atrophy, *OCB* oligoclonal bands, *Park*. Parkinsonism, *PET* positron emission tomography, *PSW* positive sharp waves, *R* right, *SOD1* superoxide dismutase 1, *SPECT* single-photon emission computed tomography, *SPEP* serum protein electrophoresis, *SVD* small vessel disease, *T4* thyroxine, *TARDBP* TAR DNA-binding protein, *TSH* thyroid stimulating hormone, *WML* white matter lesion, *WBC* white blood cells, *UL* upper limb, *Vert*. vertigo, *WM* Waldenström’s macroglobulinaemia, *WML* white matter lesion

^a^Incomplete information

Here we report three cases of a slowly progressive spastic hemiparesis consistent with Mills’ original description, with follow-up ranging from 10 to 18 years. Electrodiagnostic features and findings on MRI are described. We conclude that Mills’ syndrome, whilst part of the motor neuron disease spectrum, is a distinct nosological entity, and propose diagnostic criteria based on the published literature and our own observations. We also estimate the prevalence of this rare syndrome.

## Materials and methods

### Ethics statement

Most investigations were carried out as part of routine clinical care. Studies of motor cortical evoked potentials (MEP) and coherence were approved by the local NHS research ethics committee (approval number 08/H0908/3) and conformed to the Declaration of Helsinki. All participants provided written informed consent.

### Nomenclature

We use the term motor neuron disease (MND) to refer to the disease entity, and the term ALS to describe the specific phenotype involving a combination of upper and lower motor neuron dysfunction in the limbs.

### Patients

Three patients conforming to Mills’ original description of idiopathic progressive spastic hemiparesis were identified from the regional motor neuron disorders clinic. The referral population of this clinic includes the counties of Tyne & Wear, Northumberland, Durham and Cumbria, and encompasses 2.43 million people according to 2011 census data [24].

#### Patient 1

A 57-year-old right-handed female presented with a 4-year history of right leg weakness with equinovarus, and a reduction in grip strength in the right hand. Weakness was such that she had to lift her right leg in and out of her car with her hands. The following year, she began to experience numbness in the right hand as well as low back pain and urinary urgency. A course of intravenous methylprednisolone provided no benefit. Her condition slowly progressed but remained unilateral after 18 years, with no evidence of bulbar dysfunction. There have been no persistent sensory symptoms, though she has complained of cold extremities and acrocyanosis.

The patient was an ex-smoker. Her only past medical history of note was of curative (local) treatment for ductal breast carcinoma (11 years after onset of neurological symptoms). There was no family history of neurological disease.

##### Physical examination (initial presentation)

The gait was spastic and hemiparetic, but ambulation was unaided. There was a pyramidal catch in the right upper limb and obvious spasticity in the right lower limb. Mild pyramidal weakness (Medical Research Council (MRC) grade 4) and hyperreflexia were noted in the right upper and lower limb.

##### Physical examination (10 years after presentation)

There was an asymmetrical spastic paraparesis, worse on the right, and requiring a frame to ambulate. There were early flexion contractures of the fingers in the right hand with marked hypertonia in the right upper and lower limbs. Pyramidal weakness was noted in the right upper (MRC grade 4 proximally and grade 3 distally) and lower limb (MRC grade 3). Pathological hyperreflexia was now also evident in the left lower limb, but the left plantar response was flexor whereas the right was extensor.

##### Physical examination (12 years after presentation)

The patient had begun using a wheelchair after fracturing the right radius and ulna in a fall, and had been catheterised due to impaired mobility. She had evolved significant amyotrophy in the right hand and forearm.

#### Patient 2

In this right-handed female, left leg pain and weakness first became apparent at the age of 63. Her symptoms progressed; after 6 years she could no longer work and she began experiencing pain and weakness in the left arm. After a further 2 years she noted an unsteady gait and difficulty walking, as well as some urinary urgency. There was very little subjective progression in the subsequent 4 years.

Three years after onset, she developed vertigo and was diagnosed with left vestibular failure, and 1 year later she suffered a sixth nerve palsy, which subsequently resolved. Pregabalin effectively controlled pain in her left arm and leg. There was no relevant family history.

##### Physical examination (4 and 8 years after presentation)

The gait was unsteady and broad-based. There was subtle weakness of the left upper limb (MRC grade 4 +) and the left hand was clumsy, with more obvious pyramidal weakness in the left lower limb (MRC grade 4). Tendon reflexes were symmetrically brisk throughout, with bilateral flexor plantar responses. There was hyperalgesia and allodynia in the left upper and lower limbs. No amyotrophy or fasciculation was evident.

#### Patient 3

This right-handed retired male noticed progressive weakness in the right leg at age 60, 2 months after a low velocity road traffic accident. Over the next 3 years, his exercise tolerance reduced to half a mile with a stick, and he developed some urinary urgency and difficulty in using cutlery because of weakness in his right hand.

His past medical history was remarkable only for gout, for which he took allopurinol. There was no pertinent family history.

##### Physical examination (5 years after presentation)

During ambulation there was circumduction of the right lower limb with mild right equinovarus. There was pyramidal weakness in the right upper (MRC grade 4) and lower limb (MRC grade 4 proximally, grade 2 distally). Tendon reflexes were pathologically brisk on the right, with an extensor plantar response on that side.

##### Physical examination (10 years after presentation)

The weakness in the right upper and lower limb had increased, but there was no amyotrophy or fasciculation. Results of investigations are summarised in Tables 2 and 3. MRI scans before 2012 were performed on a 1.5 T system. Subsequent MRI scans were acquired using a 3 T system. None of the patients were treated with riluzole.

**Table 2:** Laboratory investigations

	Biochemistry	Haematology	Immunology	Virology	CSF
Patient 1	U&E NAD Ca^2+^/P/ALP NAD TSH/T4 NAD CK NAD CRP < 5 mg/l Cholesterol 4.2 mmol/l	FBC NAD Clotting NAD ESR 2 mm/h B12 305 ng/l (170–700)	SPEP NAD Auto-Ab NAD	–	Protein 0.41 g/l (< 0.4) WBC 1/mm^3^ OCB absent
Patient 2	U&E NAD Ca^2+^/P/ALP NAD TSH/T4 NAD CK NAD CRP < 5 mg/l Cholesterol 4.2 mmol/l	FBC NAD Clotting NAD ESR 2 mm/h	–	–	Protein 0.29 g/l (< 0.4) WBC < 1/mm^3^ OCB absent
Patient 3	U&E NAD Ca^2+^/P/ALP NAD TSH/T4 NAD CK NAD CRP < 5 mg/l Cholesterol 5.3 mmol/l	FBC NAD Clotting NAD APA NAD ESR 2 mm/h B12 225 ng/l (170–700)	SPEP NAD Auto-Ab NAD	HIV 1 + 2 (-)	Protein 0.7 g/l (< 0.4) WBC < 1/mm^3^ OCB matched

*ALP* alkaline phosphatase, *APA* antiphospholipid antibodies, *Auto-Ab* auto-antibodies (extractable nuclear antibodies, nuclear antibodies, rheumatoid factor, gastric parietal cell antibodies, mitochondrial antibodies), *Ca*^*2+*^ calcium, *CK* creatine kinase, *CRP* C-reactive protein, ESR erythrocyte sedimentation rate, FBC full blood count, *NAD* no abnormality detected, *P* phosphate, SPEP serum protein electrophoresis, *TSH* thyroid stimulating hormone, *T4* thyroxine, *U&E* urea and electrolytes, *WBC* white blood cells

**Table 3:** Neurophysiological findings

	Sex	Age at onset (years)	NCS/EMG		SEP		VEP		MEP		Beta-band IMC	
			Time since onset (years)	Result	Time since onset (years)	Result	Time since onset (years)	Result	Time since onset (years)	Result	Time since onset (years)	Result
Patient 1	F	53	4	NAD	14	NAD	14	NAD	14	R UL/LL + L UL: ↑CMCT (L LL: NT)	14	L UL: absent (R UL/LL: NT as unable to perform task)
			5	NAD								
			14	NAD								
			16	Fib/PSW R FDI/APB/EDC FP R FDI/ADM/APB/EDC								
Patient 2	F	63	6	NAD	10	NAD	10	NAD	9	L UL/LL: ↑CMCT R UL/LL: NAD	9	L UL/LL: absent R UL/LL: NAD
			10	NAD								
Patient 3	M	60	3	Fib R FDI PSW R TA Mild neurogenic R UL/LL	3	NAD	3	NAD	6	R UL/LL: ↑CMCT L LL: NAD (L UL: NT)	6	R UL/LL: absent L LL: absent (L UL: NT as unable to perform task)
			4	Fib R FDI PSW R TA Mild neurogenic R UL/LL								
			5	Mild chronic neurogenic R UL No Fib/PSW								
			6	Mild chronic neurogenic R UL No Fib/PSW								

*ADM* abductor digiti minimi, *APB* abductor pollicis brevis, ↑*CMCT* increased central motor conduction times, *EDC* extensor digitorum communis, *FDI* first dorsal interosseous, *Fib* fibrillation potentials, *FP* fasciculation potentials, *IMC* intermuscular coherence, *L* left, *NAD* no abnormality detected, *NT* not tested, *PSW* positive sharp waves, *R* right, *TA* tibialis anterior

### Neurophysiology

#### Evoked potentials

For somatosensory and visual evoked potentials (SEP, VEP), results were compared to local normative data. For MEP, results were compared to a previously published normative dataset [9].

#### Intermuscular (EMG–EMG) coherence

Coherence was estimated from electromyography (EMG) recordings performed during a precision grip task (upper limb) or a foot rocking task (lower limb). For the upper limb, coherence spectra were calculated between extensor digitorum communis (EDC) and first dorsal interosseous (FDI), as well as between flexor digitorum superficialis (FDS) and FDI; for the lower limb, they were computed between medial gastrocnemius (MG) and extensor digitorum brevis (EDB), and between tibialis anterior (TA) and EDB. Detailed methods were published previously [12, 16].

### Literature review

We searched Scopus, Ovid and Pubmed for publications on “Mills syndrome” in English or German, and reviewed the reference lists of the papers retrieved. Publications in other languages, including a French series of three patients with Mills’ syndrome [17], were therefore not included in the review.

## Results

Table 2 summarises the results of laboratory investigations. In one patient, the CSF showed a mildly elevated protein level and matched oligoclonal bands; serum and urinary protein electrophoresis was normal. All remaining results, including CSF parameters in the other two patients, were unremarkable.

**Fig. 1:**
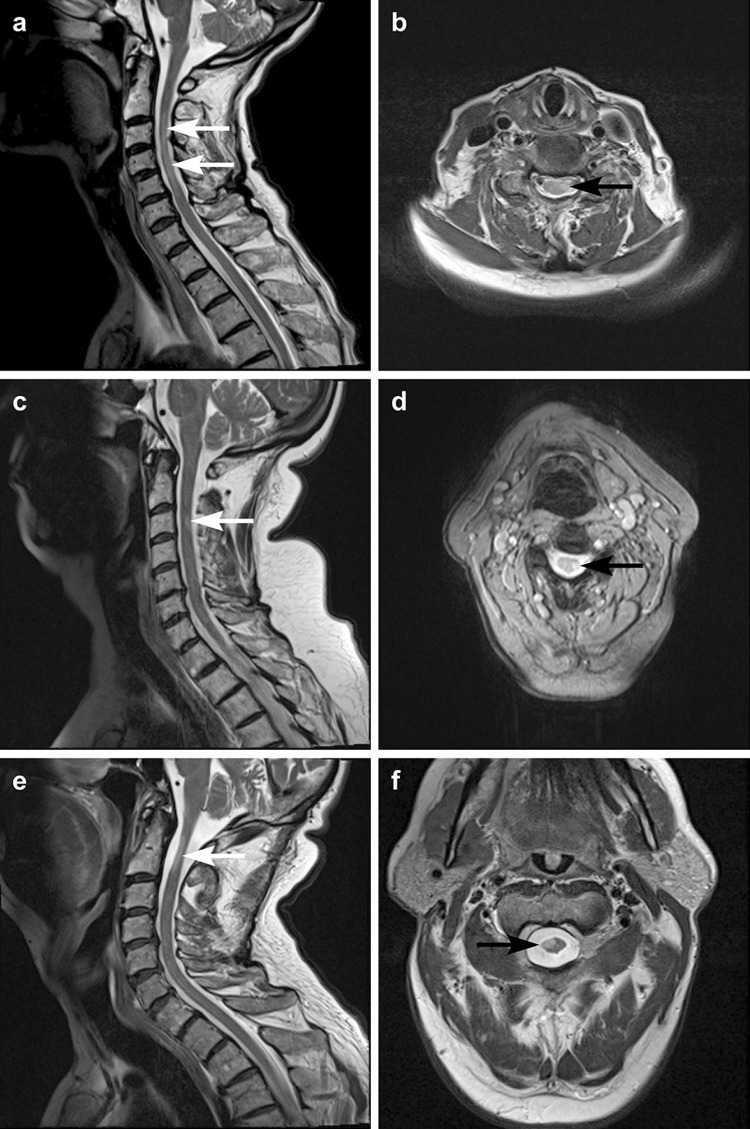
MRI findings: sagittal and axial T2 weighted images of the three patients. **a, b** Patient (1) T2 hyper-intensity in the left lateral aspect of the cervical cord at C3–C5 vertebral levels. **c, d** Patient (2) focal T2 hyper-intensity involving the left lateral aspect of the cord at C3/4 level. **e, f** Patient (3) T2 hyper-intensity involving the right anterolateral aspect of the cord at C1/2 level. All three cases have signal abnormality involving the cervical cord which is eccentric in location and involves more than one vertebral level in length

MRI brain was unremarkable in all cases. Findings for MRI of the cervical spine are illustrated in Fig. 1. In all three patients, sagittal and axial T2 weighted images returned hyper-intense signals from the lateral corticospinal tract at the cervical level, ipsilateral to the symptomatically weak limbs. In patient 1, the T2 hyper-intensity was in the left lateral aspect of the cervical cord at the C3 to C5 vertebral levels (Fig. 1a, b). In patient 2, the focal T2 hyper-intensity involved the left lateral aspect of the cord at C3/4 levels (Fig. 1c, d). In patient 3, the hyper-intensity involved the right anterolateral aspect of the cord at the C1/2 level (Fig. 1e, f). All three cases demonstrated signal abnormality involving the cervical cord, which was eccentric in location and involved more than one vertebral level in length.

The results of neurophysiological investigations are summarised in Table 3. Patient 1 did not show any abnormalities on nerve conduction studies (NCS) and EMG until 16 years after symptom onset. Those abnormalities included profuse fibrillation potentials, positive sharp waves and fasciculation potentials, together with unstable neurogenic voluntary motor unit action potentials in forearm and intrinsic hand muscles of the right upper limb, consistent with possible ALS according to Airlie House/Awaji criteria. Patient 2 exhibited no abnormality on NCS/EMG over a period of 12 years. Patient 3 showed evidence of mild neurogenic change with evidence of spontaneous activity in an intrinsic hand muscle and lower limb muscles 3 and 4 years after onset, but when examined subsequently only mild chronic neurogenic changes were found without evidence of progression.

**Fig. 2:**
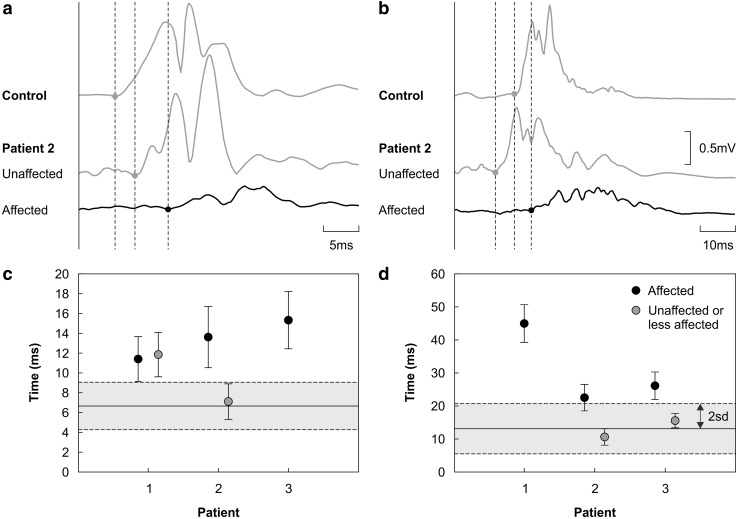
Motor evoked potentials (MEP) **a, b** Examples of average rectified MEP recorded from distal upper limb (first dorsal interosseous) and lower limb (extensor digitorum brevis) muscles. The top traces plotted in grey illustrate MEP from a healthy age-matched control subject. The lower traces are MEP recorded from patient 2 on the clinically affected and unaffected sides. Peripheral motor conduction times were removed and traces then aligned. Apparent latency differences therefore represent differences in central motor conduction times (CMCT). Vertical dashed lines indicate MEP onsets. Note that a shorter timebase has been used in **a**; the voltage scale bar applies to all MEP shown. **c, d** Central motor conduction times (CMCT) measured in the upper limb (first dorsal interosseous) and lower limb (extensor digitorum brevis) of each patient. Each data point plots the average of ten individual CMCT measurements taken from unrectified MEP. Error bars represent the standard deviation of the mean. The grey boxes delineate the range within two standard deviations of the mean (solid line) derived from normative CMCT data [9]

VEP and SEP were normal but MEP were abnormal in all patients. In patients 2 and 3, MEP were only abnormal in the symptomatic limbs, whilst patient 1 also showed abnormal MEP in the asymptomatic left upper limb (Fig. 2).

**Fig. 3:**
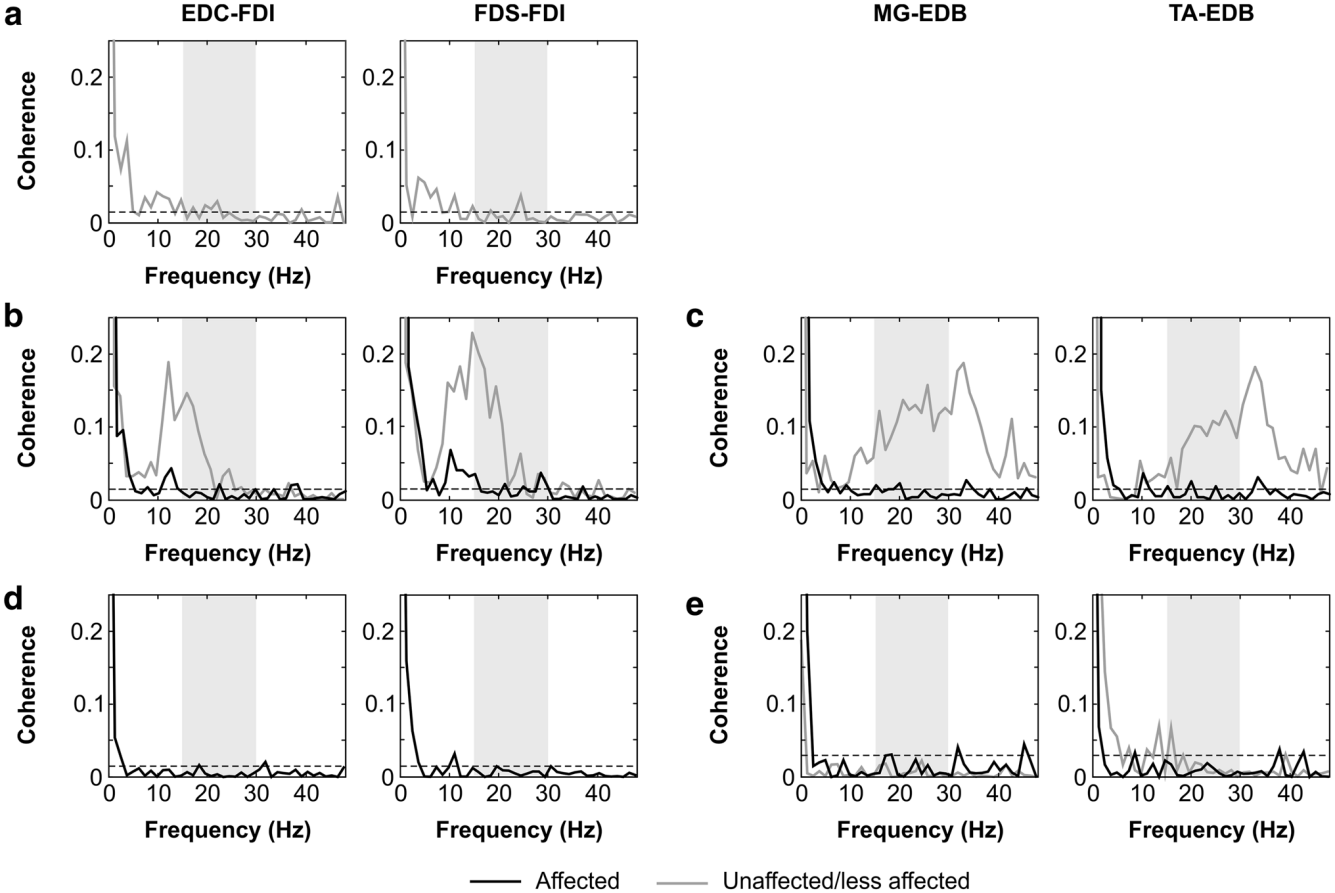
Intermuscular (EMG–EMG) coherence spectra. Coherence spectra from patients 1 (**a**), 2 (**b, c**) and 3 (**d, e**). Muscle pairs are indicated above each column (extensor digitorum communis (EDC), first dorsal interosseous (FDI), flexor digitorum superficialis (FDS), medial gastrocnemius (MG), extensor digitorum brevis (EDB), and tibialis anterior (TA)). Measurements from the affected limbs are plotted in black, whilst those from the unaffected limbs are plotted in grey. The shaded boxes demarcate the beta-band frequency range of 15–30 Hz. Dashed horizontal lines indicate the 95% significance level. In affected limbs, beta-band coherence was non-significant or borderline in all patients (patient 1 was too weak to perform the upper or lower limb tasks on the affected side). In unaffected limbs, beta-band coherence was retained in patient 2 but absent in patients 1 and 3

We also measured intermuscular coherence (Fig. 3). In patients 1 and 2, the limbs with absent coherence matched those with abnormal MEP. Interestingly, there was no significant coherence in any of the muscle pairs tested in patient 3, including in the left lower limb which was asymptomatic, normal on examination, and had shown normal MEP.

## Discussion

Here we present one of the largest, most clinically homogeneous case series of idiopathic, slowly progressive, ascending hemiparesis since Mills’ original description [21]. Unlike most of the previously published case reports (Table 1), the cohort of patients included in this series came from a single geographically defined referral population. Patients underwent prolonged follow-up with repeated clinical investigations, including MRI and clinical neurophysiology. Only one of our patients progressed to a syndrome compatible with ALS. As a consequence, we have been able to better define this clinical entity.

Only 23 cases of ‘pure’ Mills’ syndrome (if one excludes cases in which alternative diagnoses were confirmed or are likely) have been reported in the English or German language literature (Table 1) [3, 6, 8, 10, 19, 20, 22, 33], with an average length of follow-up of 16 years (range 1.5–35 years). Clinical features in our case series are consistent with those described previously, and mean follow-up was similar at 13 years (range 10–18). Combining data from our series with the 23 previously published cases (total *n* = 26), the mean age of onset was 44 (range 6–68), mean follow-up was 11 years (range 1.5–35), the M:F ratio was 13:11 and symptoms started in one leg in two-thirds of cases.

Two of our patients complained of urinary urgency (patient 1 and 3). This has only been reported in one previous case [14]. Patient 1 also complained of acrocyanosis and patient 2 of mild pain in the affected limb. Sensory symptoms, including limb pain, were described in the original series [22] and acrocyanosis was reported in a separate case, though the latter was attributed to multiple lacunar infarcts [6]. Patient 1 developed amyotrophy and thus an ALS-like phenotype after 16 years, highlighting the spectral nature of motor neuron disorders. This contrasts with a previous case where unilateral amyotrophy developed gradually alongside spasticity over 12 years before presentation [27]; in a further case, unilateral atrophy was noted at presentation, 10 years after symptom onset [11]. No amyotrophy was reported for the remaining past cases. Whilst mild but noticeable amyotrophy can occur as a consequence of disuse, particularly in a disease running a protracted course, the extent of amyotrophy in patient 1 was greater than could be ascribed to this.

Patient 3 noticed symptoms a few weeks after recovering from a low velocity road traffic accident. A similar temporal relationship to trauma was noted in a previous case report, in which paresis began developing soon after the patient had sustained open fractures of the same limb [28]. For MND, a potential link with preceding trauma has long been an area of controversy. Whilst some studies have reported a significantly elevated risk for at least 1 year after significant head or limb trauma [5, 32], given the known delays between presymptomatic disease onset, weakness and ultimately diagnosis, an aetiopathogenic role for trauma in MND seems biologically implausible [1]. Instead this association is more likely to reflect a greater risk of falls in incipient MND. We consider it likely that the association between Mills’ syndrome and trauma is also coincidental.

An important diagnosis to consider in an evolving hemiparesis, paraparesis or tetraparesis is the central demyelinating disorders including multiple sclerosis. Notably, in our case series, there was no evidence of these on CSF studies, VEP or SEP.

Whilst acute demyelination is distinguishable from gliosis on MRI T2 or FLAIR sequences because of oedema (and enhancement with contrast), the chronic changes of demyelination are not. In multiple sclerosis, demyelinating lesions, particularly those within the spinal cord, do not typically respect anatomical boundaries, have indistinct borders (for example, see axial MRI sections from patients 2–4 in the figure published in [29]) and are confined to one vertebral segment [4]. By contrast, the MRI signal changes observed in our cohort were confined strictly to the lateral funiculus, with defined, demarcated borders, and extended for two or more vertebral segments on sagittal images. This latter feature is atypical for multiple sclerosis (or solitary sclerosis) and therefore suggests alternative aetiologies including Wallerian degeneration and gliosis [2].

Electrophysiological examination is important in helping to define alternative progressive upper motor neuron syndromes [15, 26]. A complete set of NCS, needle EMG and evoked potentials was reported for only two of the reviewed previous cases [13, 33] but is provided for all cases presented here. Whilst needle EMG, SEP and VEP are important in excluding other diagnoses, MEP can confirm the presence of corticospinal tract disease. MEP have been reported in three previous cases [28, 30, 33] and showed unilaterally prolonged central motor conduction times and—in one case—borderline central motor conduction times in the contralateral lower limb. In our cohort, abnormalities in central motor conduction times were also unilateral, except in patient 1, where MEP were also delayed in the asymptomatic contralateral upper limb when tested 14 years after onset (Fig. 2; Table 3).

It has been suggested that intermuscular coherence analysis is more sensitive than MEP in detecting corticospinal tract dysfunction [12]. Intermuscular coherence is mediated by an efferent–afferent feedback loop and thus depends on the integrity of both the corticospinal tract and afferent pathways [35]. All patients who were able to perform the coherence task on the (most) affected side had abnormal coherence on that side, which could only be attributed to disease in the corticospinal tract because sensory NCS and SEP were normal. Patient 1 had abnormal coherence in the asymptomatic upper limb, consistent with the results of MEP. In patient 3, the upper limbs exhibited unilaterally abnormal MEP but bilaterally abnormal coherence. This suggests the presence of subclinical corticospinal tract disease on the clinically unaffected side, and supports the notion that intermuscular coherence is more sensitive than MEP in detecting corticospinal tract disease.

Similar to patient 1, some past cases exhibited upper motor neuron signs contralateral to the side of onset [14, 27], and post-mortem histology, which was performed in two previous cases, demonstrated bilateral degeneration of the corticospinal tract [11, 23]. Together with the electrophysiological evidence of bilateral corticospinal tract involvement, these features might be viewed as consistent with PLS. However, if diagnostic criteria for PLS [26] are strictly applied these require symmetry at presentation (or within 4 years) and permit pseudobulbar palsy as a first presentation, which has never been described in Mills’ syndrome. Betz cells in the precentral gyrus are absent or reduced in PLS but are preserved in Mills’ syndrome, thus further supporting the notion that Mills’ syndrome is a distinct entity. The absence of positivity for TAR DNA-binding protein 43 in a recent case also distinguishes Mills’ syndrome from MND [11].

**Fig. 4:**
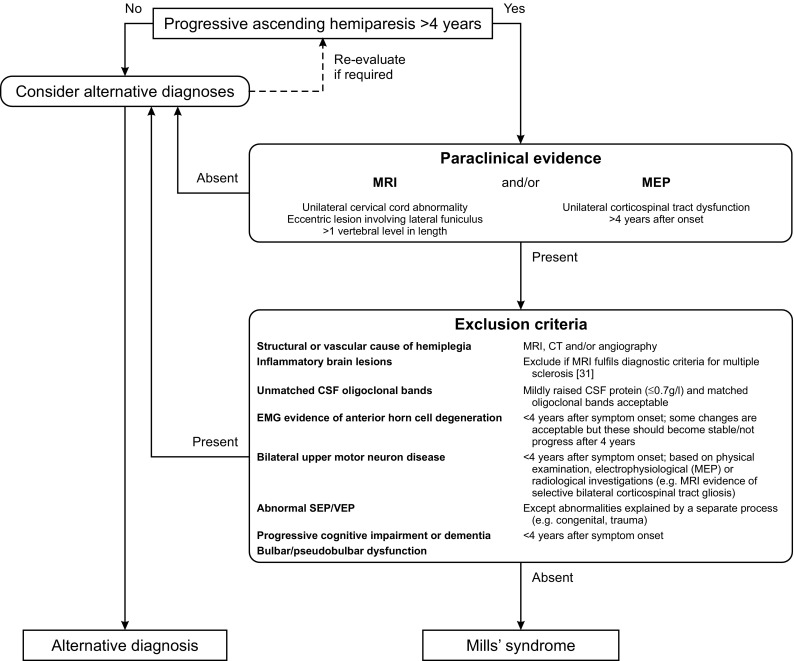
Proposed diagnostic algorithm for Mills’ syndrome A diagnosis of Mills’ syndrome requires a progressive ascending hemiparesis for at least 4 years, paraclinical evidence for a unilateral corticospinal tract lesion based on MRI or motor evoked potentials (MEP), and the absence of a range of exclusion criteria

Our proposed diagnostic algorithm for Mills’ syndrome, encompassing features based on previously published criteria for PLS [26], upper motor neuron-dominant ALS [15] and ALS [7], is outlined in Fig. 4. If our criteria are satisfied, based on our experience and review of the literature, a number of statements can be made with regard to prognosis. First, no patient with the condition has yet died directly from Mills’ syndrome. Second, the shortest delay between symptom onset and the appearance of features typical of ALS is 12 years and the longest follow-up without evidence of progression to ALS is 26 years to date. Finally, disability accumulates slowly. In our cohort, only one patient became dependent on a wheelchair after 14 years of symptoms.

Our referral population encompasses 2.43 million people, according to 2011 census data [24], leading to a point estimate of 1.2:1,000,000 for the prevalence of Mills’ syndrome. This is likely to be an underestimate, mainly because patients with Mills’ syndrome in primary or secondary care are more likely to receive alternative, more common diagnoses such as stroke. However, we hope that the provision of clear diagnostic criteria will improve case ascertainment and allow more accurate prevalence estimates in the future.
